# Pregnancy and birth in HIV seropositive women - present stage results

**DOI:** 10.1186/1471-2334-13-S1-O34

**Published:** 2013-12-16

**Authors:** Mihai Mitran, Carmen Georgescu, Mariana Mărdărescu, Bogdan Marinescu, Andreea Velişcu, Ioana Roşca, Marcela Șerban

**Affiliations:** 1 Clinical Hospital of Obstetrics and Gynecology “Prof. Dr. Panait Sârbu”, Bucharest, Romania; 2 National Institute for Infectious Diseases "Prof. Dr. Matei Balş", Bucharest, Romania; 3 The Institute for Mother and Child Protection “Alfred Rusescu”, Bucharest, Romania

## Background

Since 1992, the Clinical Hospital of Obstetrics and Gynecology “Prof. Dr. Panait Sârbu”, Bucharest has been the medical unit to consult, record and monitor pregnancies, births and miscarriages associated with transmittable diseases - including HIV positive patients - from Bucharest and the surrounding counties, as the hospital had the necessary professional expertise and intrahospital networks. In time, within the “Prof. Dr. Panait Sârbu” Clinic, we have systematized and implemented an obstetric protocol for the prevention of HIV vertical transmission.

## Methods

The aim of this protocol has been to reduce the HIV vertical transmission rate from 30-35%, the value in 2000, to 2-3%, the rate recorded in the European developed countries.

Starting from the primordial objective to decrease mother-to-child vertical transmission rate, we established the cesarean section as the method of delivery for all HIV positive women, at 37-38 weeks, on intact membranes, outside labor. The result was a significant decrease in the number of HIV positive babies.

## Results

The results of the past 7 years have been most encouraging, with a 0 rate of vertical transmission through C-section, and a 23.63% transmission for vaginal delivery.

## Conclusion

Attentive monitoring of pregnancy by the obstetrician in cooperation with the infectious diseases specialist, delivery by C-section, complex antiretroviral therapy for both mother and child and ablactation have been the main means through which a spectacular decrease in HIV vertical transmission has been achieved.

